# Moving from neurodegenerative dementias, to cognitive proteinopathies, replacing “where” by “what”…

**DOI:** 10.1590/1980-57642020dn14-030005

**Published:** 2020

**Authors:** Ricardo Francisco Allegri

**Affiliations:** 1 Departament of Cognitive Neurology, Neuropsychology, and Neuropsychiatry, Instituto de Investigaciones Neurologicas Fleni, Buenos Aires, Argentina.; 2 Department of Neurosciences, Universidad de la Costa, Barranquilla, Colombia.

**Keywords:** Alzheimer disease, proteins, frontotemporal dementia, biomarkers, dementia, doença de Alzheimer, proteínas, demência frontotemporal, biomarcadores, demência

## Abstract

Neurodegenerative dementias have been described based on their phenotype, in relation to selective degeneration occurring in a particular neuroanatomical system. More recently however, the term proteinopathy has been introduced to describe diseases in which one or more altered proteins can be detected. Neurodegenerative diseases can be produced by more than one abnormal protein and each proteinopathy can determine different clinical phenotypes. Specific biomarkers have now been linked to certain molecular pathologies in live patients. In 2016, a new biomarker-based classification, currently only approved for research in Alzheimer’s disease, was introduced. It is based on the evaluation three biomarkers: amyloid (A) detected on amyloid-PET or amyloid- beta 42 assay in CSF; tau (T) measured in CSF as phosphorylated tau or on tau PET imaging; and neuronal injury/neurodegeneration (N), detected by total T-tau in CSF, FDG PET hypometabolism and on MRI brain scan. Results of clinical research using the ATN biomarkers at FLENI, a Neurological Institute in Buenos Aires, Argentina have, since 2011, contributed to ongoing efforts to move away from the concept of neurodegenerative dementias and more towards one of cognitive proteinopathies. Today, clinical diagnosis in dementia can only tell us “where” abnormal tissue is found but not “what” molecular mechanisms are involved.

## RESUMO.

As demências neurodegenerativas foram descritas com base em seu fenótipo, em relação à degeneração seletiva que ocorre em um sistema neuroanatômico específico. Mais recentemente, no entanto, o termo proteinopatia foi introduzido para descrever doenças nas quais uma ou mais proteínas alteradas podem ser detectadas. As doenças neurodegenerativas podem ser produzidas por mais de uma proteína anormal e cada proteinopatia pode determinar diferentes fenótipos clínicos. Biomarcadores específicos já foram associados a certas patologias moleculares em pacientes vivos. Em 2016, uma nova classificação baseada em biomarcadores, atualmente aprovada apenas para pesquisas na doença de Alzheimer, foi introduzida. É baseado na avaliação de três biomarcadores: amiloide (A) detectado no ensaio amiloide-PET ou amiloide-beta 42 no LCR; tau (T) medida no LCR como tau fosforilada ou em imagem de tau PET; e lesão/neurodegeneração neuronal (N), detectada por T-tau total no LCR, hipometabolismo FDG PET e pela ressonância magnética. Os resultados de pesquisas clínicas usando os biomarcadores ATN no FLENI, um Instituto Neurológico de Buenos Aires, Argentina, desde 2011, contribuíram para os esforços contínuos para se afastar do conceito de demência neurodegenerativa e mover-se mais em direção às proteinopatias cognitivas. Hoje, o diagnóstico clínico da demência só pode nos dizer “onde” o tecido anormal é encontrado, mas não “quais” mecanismos moleculares estão envolvidos.

## INTRODUCTION

The main pathophysiological mechanism underlying Alzheimer’s disease (AD) involves extracellular amyloid deposits and neurofibrillary degeneration secondary to abnormal tau protein hyperphosphorylation. AD is present many years before symptoms develop. Bateman et al. for example, detected amyloid deposits over 20 years, and neurofibrillary degeneration over 10 years, prior to the onset of clinical symptoms.^1^ Many neurodegenerative disorders, including AD, frontotemporal dementia (FTD), Lewy body dementia, and Huntington’s disease are considered today to be proteinopathies associated with aggregation and accumulation of misfolded proteins.

Prior to the development of the AD biomarker, clinical diagnosis was identified as either possible or probable, and definite diagnosis needed to be confirmed by post-mortem brain tissue histopathology.^2^ The discovery of AD biomarkers gave rise to a new paradigm in relation to degenerative dementias. The biomarker assay allows *in vivo* assessment of pathophysiological disease traits. Current biomarkers used in clinic for AD include:

- Aβ1-42, total tau and phosphorylated tau assay in cerebrospinal fluid (CSF);
- structural neuroimaging studies such as brain magnetic resonance imaging (MRI) and hippocampal volume analysis;
- functional neuroimaging of metabolic activity such as fluorodesoxyglucose (FDG ) positron emission tomography (PET);
- protein-identifying neuroimaging using amyloid and tau PET.^3^

## CLINICAL DIAGNOSIS IN DEMENTIA TELL US “WHERE” BUT NOT “WHAT”

Neurodegenerative dementias were described the early twentieth century based on phenotypic manifestations secondary to the involvement of different central nervous system areas (extrapyramidal, cerebellar, memory or behavioral etc.). Later, they were defined as diseases resulting from systematic degeneration of different neuroanatomical pathways. For example, Alzheimer was considered to be the result of cognitive impairment caused by degeneration of the parietotemporal cortex, Pick as a behavioral disease caused by frontotemporal lobar degeneration, and Lewy Body Dementia as secondary to cortical and extrapyramidal degeneration.

Neurodegenerative dementias (AD, frontotemporal dementia etc.) were classified by clinical phenotype. Over the past 10 years, however, clinical forms of AD have been further subdivided into typical (hippocampal amnesia) and atypical (visuospatial, logopenic aphasia, and frontal variant),^4^ which best describe the alternative presentations, some of which are more, and some less frequent.^5^ Another neuropathological discovery was that typical amyloid and tau signature lesions were present in early-onset dementia associated with familial or sporadic Alzheimer. However, in patients with late-onset AD, although amyloid deposits and tau pathology were also detected, other additional abnormalities were diagnosed including TDP-43 and alfa-synuclein deposits as well as vascular disease.^6^

FTD was first described as a behavioral syndrome causing apathy and disinhibition. Currently, it also includes semantic and non-fluent variants of primary progressive aphasia (PPA) and, in some patients, it has been associated with amyotrophic lateral sclerosis. Neuropathology findings are diverse and include tau pathology, TDP43, fused-in sarcoma protein (FUS ), as well as amyloid deposition.^7^ In the last year, a study found that 25% of typical amnestic hippocampal AD patients did not present AD neuropathology, but had TDP-43 deposition, a population for which the term Limbic-predominant age-related TDP-43 encephalopathy (LATE) was proposed.^8^

**Figure 1.**
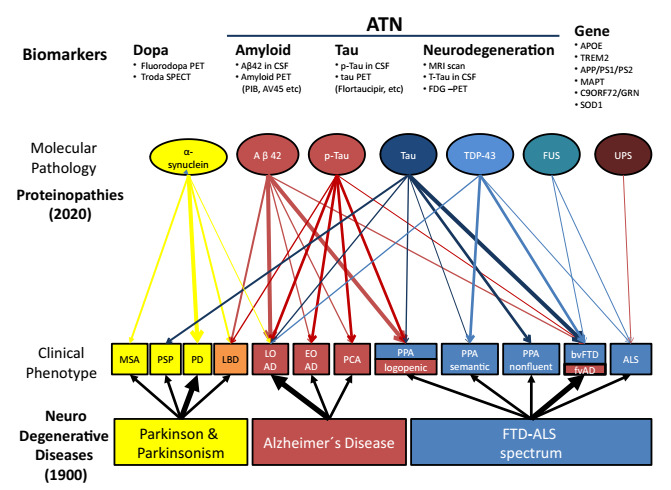
Proteinopathies in neurodegenerative dementia.

Ultimately, different “neurodegenerative diseases” can be produced by different proteinopathies, each determining varying clinical phenotypes (Figure 1).

MSA: multi systemic atrophy; PSP: progressive supranuclear palsy; PD: Parkinson disease; LBD: Lewy Body dementia; LOAD: late onset Alzheimer; EOAD: early onset Alzheimer disease; PCA: posterior cortical atrophy; PPA: progressive primary aphasia; bvFTD: behavioral variant frontotemporal dementia; fvAD: frontal variant Alzheimer disease; ALS: amyotrophic lateral sclerosis; TDP-43: TAR DNA-binding protein 43; FUS: Fused-in Sarcoma protein; UPS: Ubiquitin proteasome; APOE: apolipoprotein E; TREM2: triggering receptor expressed on myeloid cells 2; APP: amyloid precursor protein; PS1: presenilin 1; PS2: presenilin 2; MAPT: microtubule-associated protein tau; GRN: progranulin, SOD1: superoxide dismutase-1; ATN: amyloid, tau, neurodegeneration.

Research in neurodegenerative diseases cannot rely on clinical phenotypes alone, as was the case with classic diagnostic criteria;^2^ a multimodal approach is required, identifying underlying proteinopathies responsible for clinical symptoms.

Clinical phenotypes reflect neuroanatomical system involvement, i.e., ‘where’ the disease is located. If a patient has PPA, the language system in the left hemisphere is affected, if it is non-fluent, the left frontal lobe is the source, if it is semantic, then the left anterior temporal lobe is the site of origin.^9^ An amnesic syndrome of the hippocampal profile affects the hippocampus; the dysexecutive syndrome affects the frontal lobe; and the visuospatial syndrome, the posterior parietal cortex.^4^ One form of dysexecutive syndrome (frontal degeneration) has been linked to tauopathy, TDP-43 or even amyloidopathy,^4^ and amnesic hippocampal syndrome may be caused by amyloidopathy or TDP-43.^8^ Current clinical dementia diagnoses recognize ‘where’ the pathology may be found, but not ‘what’ the underlying pathophysiology is.

Degenerative dementias need to be investigated based on molecular findings. Alzheimer’s research cannot be referred to today without including biomarker results, a situation that is challenging from the public health perspective, particularly in developing countries such as those of the Latin American region.

## EMERGING BIOMARKERS IN PROTEINOPATHIES

Since the original description of diagnostic criteria by Mc Khan et al. in 1984, diagnosis of AD was considered to be either probable or possible, with a definite diagnosis established only by neuropathology examination after the death of a patient.^2^ More recently, ‘biomarkers’ indicating underlying molecular pathology in living patients have been discovered. Different clinical biomarkers for AD are now under investigation, and several more in other proteinopathies.^3^

Clinical symptoms in AD are not necessarily secondary to amyloid deposits; they do not correlate with levels or sites of accumulation, but respond instead to neuronal damage secondary to neurofibrillary degeneration. Amyloid deposition begins in the frontal lobe and precuneus areas, and tau pathology in the hippocampus, progressing to parieto-temporal association areas. It has now been established that tau progression follows neuronal pathways. Different authors have observed similarities between the passage of tau from one neuron to another and mechanisms occurring in prion diseases, with the difference that the transmission of tau does not occur between actual patients.^10^

Misdiagnosis rates were high when based on phenotype alone, as described by Mc Khan criteria.^2^ Later, Dubois et al. defined Prodromal AD, the first diagnostic criteria for AD research based on the presence of a hippocampus amnesic phenotype plus one positive AD biomarker.^11^ However, the criteria were difficult to apply, since they only included patients presenting amnesic phenotypes with mild cognitive impairment, and biomarkers were restricted to amyloid and tau.^11^ In 2011, the NIA (National Institute on Aging) and the AA (Alzheimer’s Association)^12^ published wider, more inclusive criteria based on AD biomarkers, establishing disease stages as a continuum from pre-symptomatic AD, for mild cognitive impairment followed by dementia. In relation to biomarkers, three sub-stages were described: amyloid only, amyloid plus neurodegeneration, and amyloid plus neurodegeneration as well as subtle clinical changes. In 2014, Dubois et al. expanded diagnostic criteria for typical (amnestic-hippocampal) and atypical forms (cortical posterior atrophy - CPA, PPA, frontal, and Down variants), prioritizing amyloid markers in the diagnosis.^4^

Finally, Jack et al.^13^ launched a new biomarker-based biological A/T/N (Amyloid/Tau/ Neurodegeneration) classification, where “A” refers to the presence of β-amyloid biomarker (detected in amyloid PET or assaying CSF Aβ42 level); ‘T’, the value of a tau biomarker (measured in CSF using a phosphorylated tau assay, or tau PET); and ‘N’ to biomarkers for neurodegeneration or neuronal injury (evaluated on [18F]-fluorodeoxyglucose-PET, structural MRI atrophy, or measurement of total tau in CSF). This classification provides both pathophysiological categorization and clearer prediction of patient outcome.

## FLENI EXPERIENCE: MULTIMODAL APPROACH AND INTERNATIONAL NETWORK

Fifteen years ago, Fleni began applying a multimodal approach to study patients with cognitive impairment. Five interrelated platforms were introduced to workup patients: clinical (cognitive and behavioral); neuroimaging (3.0-T brain MRI scan, amyloid, and FDG PET-CT); biochemical (CSF AD amyloid-B1-42markers, total tau protein and tau phosphorylated at threonine position 181, pTau-181); genetic; and brain banking.^14^

In 2011, Fleni joined the Alzheimer’s Disease Neuroimaging Initiative (ADNI), a worldwide network of Alzheimer centers originally started in the US by the National Institutes of Health (NIH ) to harmonize existing platforms, and became the first center from Latin America to participate.^15^ The Argentine ADNI^16^ recruited 60 participants, 30 patients with mild cognitive impairment, 15 with Alzheimer’s dementia, and 15 normal control subjects, to better characterize AD in Argentina.^14^ Since the beginning, the Arg-ADNI has established strong ties with different research sites worldwide to improve recruitment and harmonize clinical and biomarker data management.

Surace et al., reported in 2013, results of AD biomarker assay in CSF and application of findings to better discriminate AD from FTD, as well as to predict progression from mild cognitive impairment (MCI) to AD.^17^ Significant differences between groups with AD and FTD were observed in biomarker levels and ratios. When the group with MCI was analyzed and sub-divided based on clinical progression to AD over time, significant differences were observed in mean values of amyloid-beta 1‒42 in those with progression (355 pg./mL) *versus* those without progression to AD (800 pg./mL). Receiver operating characteristic (ROC) curve analysis performed between groups showed biomarker cutoff values as follows: Ab42, 532.5 pg./mL (sensitivity 100%, specificity 87.5%); tau 100 pg./mL (sensitivity 84.5%, specificity 87.5%); p-tau, 26.5 pg./mL (sensitivity 69.2%, specificity 87.5%); Ab42/p-tau, 20.5 pg./mL (sensitivity 92.3%, specificity 87.5%); AD CSF profile 1.350 pg./mL (sensitivity 100%, specificity 100%).

Link between cognitive reserve and CSF Aβ1-42 levels studied in this population by Harris et al. showed significant correlation.^18^ These results support the concept that greater cognitive reserve, as evidenced by higher Aβ1-42 levels, exerts a protective role against progression to AD, in patients with MCI.^18^

In 2015, amyloid PET scan utility was studied in the clinical setting, showing agreement between 11C-PIB-PET findings and clinical diagnosis.^19^ A retrospective study including 144 patients (40 from the Argentine ADNI), divided patients into clinical categories of high or low probability of AD pathology. The former included: amnestic MCI; amnestic multi-domain MCI; dementia of Alzheimer’s Type (DAT); posterior cortical atrophy (PCA); logopenic PPA; cerebral amyloid angiopathy; as well as mixed dementia. The low clinical probability group included: normal controls; non-amnestic MCI; non-logopenic PPA; and frontotemporal dementia patients. Overall concordance between scan results and clinical diagnosis was 72.6% for high pretest probability, and 73.6% for low pretest probability. Among high pretest probability patients, 68% of a-MCI, 60% am-MCI, 76% of DAT, and 100% of logopenic-PPA, PCA, and cerebral amyloid angiopathy (CAA) patients had positive amyloid PET scans. In contrast, results in the low pretest probability group were more heterogeneous. In all, 5% of normal subjects, 33% of non-memory-MCI, 33% behavioral variant FTD (bvFTD), and 45% of PPA patients were amyloid positive. The study demonstrated the importance of detecting *in vivo* amyloid plaque deposition using molecular imaging in atypical patients, such as in cases of early-onset dementia, PCA, PPA, and non-amnesic MCI.^19^

A cross-sectional analysis of baseline data revealed links between episodic memory performance, hippocampal volume, and other biomarkers.^20^ Furthermore, the combination of recognition discrimination index and Delayed Recall test results proved useful to predict conversion from MCI to dementia.^21^ ^,^ ^22^

Findings regarding cognitive decline and rate conversion of MCI to dementia (20% in one year) of this cohort were published in 2018.^23^ Russo et al. described, also in 2018, use of a Spanish version of the Everyday Cognition scale which was more sensitive than the Functional Assessment Questionnaire (FAQ) in the evaluation of patients with MCI.^24^

**Figure 2.**
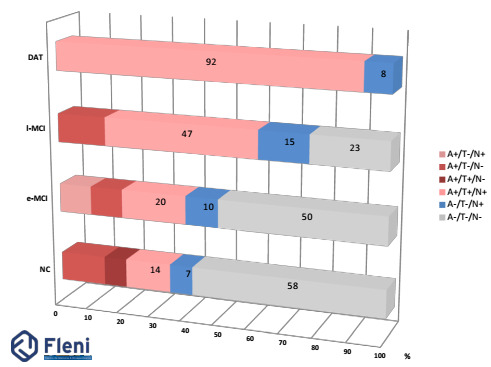
Percentage of patients with each amyloid, tau, neurodegenaration subtype in the Arg-ADNI population. In red positive amyloid and in blue SNAPs.

NC: normal controls; eMCI: early MCI; lMCI: late MCI; DAT: dementia Alzheimer type; ATN: amyloid, tau, neurodegenaration.

The new A/T/N classification by Jack et al.,^13^ ^,^ ^25^ was applied to this population.^26^ The results are shown in Figure 2.

In the search for newer and simpler biomarkers, CSF neurofilament light chain (NfL) was studied in 2019, in a cognitive clinical setting and was found to be significantly increased in MCI, FTD, and DAT patients compared to normal controls. Interestingly, ROC curve analysis showed the highest area under the curve (AUC) value when comparing CSF NfL in control *versus* FTD patients, making it a promising biomarker of neurodegeneration.^27^

In 2013 we reported one AD family with a PSEN 1 mutation (M146v) characterized by a frontotemporal phenotype^28^ and in 2020 we described a new PSEN 1 mutation (p.T119l) in another Argentine family with both early and late clinical onset AD.^29^ As part of this multimodal approach, FLENI now has a brain bank with over 120 specimens, five from Dominantly Inherited Alzheimer Network (DIAN) families.

Currently, we are using the A/T/N classification to report prognosis in the Arg-ADNI cohort. After a 5-year follow-up, the conversion rate from MCI to dementia was 85% in A+T+N+ patients and 50% in A-T-N+ patients.^26^

When describing the clinical phenotype of a neurodegenerative patient, we currently report the brain region affected by the disease, with light reference to etiology. This form of classification seems now to be an outdated neurological concept that will likely be replaced in the near future by detailed information on cognitive proteinopathy findings. This new concept will contribute to a better understanding of future AD treatments. Latin America should look for ways to access these new studies, probably developing more collaborative works between different countries.
